# New neural network classification method for individuals ancestry prediction from SNPs data

**DOI:** 10.1186/s13040-021-00258-7

**Published:** 2021-06-28

**Authors:** H. Soumare, S. Rezgui, N. Gmati, A. Benkahla

**Affiliations:** 1 The Laboratory of Mathematical Modelling and Numeric in Engineering Sciences, National Engineering School of Tunis, Rue Béchir Salem Belkhiria Campus universitaire, B.P. 37, 1002 Tunis Belvédère, University of Tunis El Manar, Tunis, Tunisia; 2 Laboratory of BioInformatics, bioMathematics, and bioStatistics, 13 place Pasteur, B.P. 74 1002 Tunis, Belvédère, Institut Pasteur de Tunis, University of Tunis El Manar, Tunis, Tunisia; 3 ADAGOS. Le Belvédère centre, 61 rue El Khartoum, El Menzah, Tunis, Tunisia; 4 College of sciences & Basic and Applied Scientific Research Center, Imam Abdulrahman Bin Faisal University, P.O. Box 1982, 31441, Dammam, Kingdom of Saudi Arabia, Imam Abdulrahman Bin Faisal University, Dammam, Saudi Arabia

**Keywords:** Artificial neural network, Dimensionality reduction, Input perturbation, Single nucleotide polymorphism, Singular value decomposition

## Abstract

Artificial Neural Network (*ANN*) algorithms have been widely used to analyse genomic data. Single Nucleotide Polymorphisms(*SNPs*) represent the genetic variations, the most common in the human genome, it has been shown that they are involved in many genetic diseases, and can be used to predict their development. Developing *ANN* to handle this type of data can be considered as a great success in the medical world. However, the high dimensionality of genomic data and the availability of a limited number of samples can make the learning task very complicated. In this work, we propose a New Neural Network classification method based on input perturbation. The idea is first to use *SVD* to reduce the dimensionality of the input data and to train a classification network, which prediction errors are then reduced by perturbing the *SVD* projection matrix. The proposed method has been evaluated on data from individuals with different ancestral origins, the experimental results have shown the effectiveness of the proposed method. Achieving up to **96.23%** of classification accuracy, this approach surpasses previous Deep learning approaches evaluated on the same dataset.

## Introduction

The human genome contains three billion of base pairs, with only 0.1*%* difference between individuals [1]. The most common type of genetic variations between individuals is called Single Nucleotide Polymorphism (*SNP*) [2]. An *SNP* is a change from one base pair to another, which occurs about once every 1000 bases. Most of these *SNPs* have no impact on human health. However, many studies have shown that some of these genetic variations have important biological effects and are involved in many human diseases [3, 4]. *SNPs* are commonly used to detect genes associated with the development of a disease within families [5]. In addition, *SNPs* can also help to predict a person’s response to drugs or their susceptibility to develop one or more particular diseases. In genetics, Genome-Wide Association Studies (*GWAS*) are observational studies that use high-throughput genotyping technologies to identify a set of genetic variants that are associated to a given trait or disease [6], by comparing variants in a group of cases with variants in a group of controls. However, this approach is only optimal for populations from the same ancestry group, as it is challenging to dissociate the variations associated with a disease from those that characterize the genetic of human populations. In this context, numerous machine learning algorithms have been used to classify individuals according to genetic differences that affect their population. Support Vector Machines (*SVM*) methods have been applied to infer recent genetic ancestry of a subgroup of communities in the USA [7] or coarse ethnicity [8]. However, *SVM* methods are very sensitive to the choice of kernel and its parameters [9]. Deep learning algorithms, such as Neural Networks have been widely used to analyse genomic data as well as gene expression data to classify certain diseases [10–20]. But, the high dimensionality of genomic data (when the number of input features is several times higher than the number of training examples) makes the learning task very difficult. Indeed, when data is composed of a large number of input features *m* for a small number of samples *n* (*n*<<*m*), the problem of overfitting becomes inevitable. In general, overfitting in machine learning occurs when a model fits well with the training data, but not fit the unseen data. The model learns details and noise in the training data, which negatively impact the performance of the model on new data. One way to avoid the problem of overfitting is to reduce the complexity of the problem by removing features that do not contribute or decrease the accuracy of the model [21]. Different techniques are used to deal with the problem of overfitting. The most well-known ones are *L*^1^ and *L*^2^ regularizations [22]. The idea of these techniques is to penalize the higher weights in the model by adding extra terms in the loss function. Another commonly used regularization technique, called "Dropout", introduced by Hinton et al. [23] consists of dropping neurons at random (in hidden layers) in each learning round. However, with such difference between the number of features versus the number of samples, it increases the problem of overfitting. To overcome this problem, dimensionality reduction techniques need to be combined with unsupervised learning methods or other data preprocessing techniques.

There are many ways to transform a high-dimensional data to low-dimensional data, Singular Value Decomposition (*SVD*), Principal Component Analysis (*PCA*)) and Autoencoder(*AE*) are the most common dimensional reduction techniques. *SVD* and *PCA* are the most popular linear dimensionality reduction techniques. Both attempt to find *k* orthogonal dimensions in an *n*-dimensional space, so that *k*<*n*. They are related to each other, but *PCA* uses the covariance matrix of the input data, while *SVD* is performed on the input matrix itself. The Autoencoder is a Neural Network that tries to reconstruct the input data from their compressed form. Indeed, the Autoencoder is used as a method of non-linear dimensionality reduction, it works by mapping an n-dimensional input data into a k-dimensional data (with *k*<*n*).

Recently, *ANN*s have been used in many works to analyse sequencing data and predict complex diseases using *SNPs* data [11, 24–29]. To analyse *SNPs* from sequences [16, 26, 30], many approaches have been proposed to deal with high dimensionality by combining dimensionality reduction techniques, such as unsupervised methods followed by supervised Neural Networks for classification [11, 13, 31–33]. For instance, Zhou et. al. [11] used a three-step Neural Network to characterise the determinants of Alzheimer’s disease. Liu et al. [34] combined Deep Neural Network with an incremental way to select *SNPs* and multiple Dropouts regularization techniques. Kilicarslan et al. [32] used a hybrid model consisting of Relief and stacked Autoencoder as dimensionality reduction technique followed by Support Vector Machines (SVM) and Convolutional Neural Networks (CNNs) for diagnosis and classification of cancer samples. Khan et al. [35] used *PCA* and Neural Network to identify relevant genes and classify cancer samples. Fakoor et al. [14] combined *PCA* with Sparse Autoencoder to improve cancer diagnosis and classification. Romero et al. [33] proposed to reduce the hyperparameters of the classification network by the use of auxiliary networks. Pirmoradi et al. citepirmoradi2020self used Deep Auto-Encoder approach to classify complex diseases from *SNPs* data. Based on our literature review, Romero et al. are the first to use Deep learning algorithms on *SNP* data for genetic ancestry prediction task. They constructed a classification network with an optional reconstruction path and proposed two auxiliary Neural Networks to predict the parameters of the first layer of the classification network and its reconstruction path respectively. They proposed several types of embedding techniques to reduce the number of free parameters in the auxiliary networks, such as *Random projection(RP)*, *Per class histogram*, *SNPtoVec*, *Embedding learnt end-to-end from raw data*.

In this work, we propose a New Classification Neural Network based on the perturbation of the input matrix. To address the problem of dimensionality, the training model is constructed in three steps followed by a test phase: (1) use *SVD* to reduce the dimension of the input data, (2) train a Multi-Layer Perceptron (*MLP*) to perform classification tasks, (3) perturb the *SVD* projection matrix in the sense to minimize the training loss function. In the test phase, the test set is multiplied by the perturbed projection matrix to evaluate the performance of the classifier.

The main contribution of this paper, is how the projection matrix is perturbed after the model is trained. This perturbation is inspired by the Targeted Attacks Method, which aims is to change the inputs so that the network classify them into any desired class [36–40]. These inputs are called Adversarial Examples. Previews works on target attacks have been used in image analysis, such as image segmentation [41], face detection [42] or image classification [43]. There are many ways of producing adversarial examples [44–46], the most commonly used one is Fast Gradient Sign Method (*FGSM*) and its variants [40, 47]. The proposed approach uses *FGSM* to perturb the input data iteratively to maximize the probability that each output sample falls into the desired class. Other variants of this method, such as Projected Gradient Descent [45], Basic Iterative Method [47], Boosting FGSM with Momentum [48] and many other gradients based methods, could be used [49–51]. For instance, the Projected Gradient Descent is considered as one of the most effective algorithms to generate adversarial samples. However, this method is too time-consuming to be used for training. FGSM is a very simple and fast method of generating adversarial examples [40]. The objective is to obtain a good representation of input features in SVD projection space, which will be obtained after calculating the perturbed input of the training data.

This work is organized as follows: the proposed method and the dataset used are described in “Material and methods” section, the obtained results are reported in “Results” section and the experiments are discussed in “Discussion” section.

## Material and methods

The proposed approach uses *SVD* to reduce the number of free parameters of the classification network. However, others dimensionality reduction techniques could be used. For instance, Per class histogram method [33] is a very simple dimensionality reduction technique. The idea of this technique is to represent each feature (*SNP*) in the input data by 3 possible values, corresponding to the proportion of ethnic groups having as genotype 0, 1 or 2 respectively. This produces a projection matrix of size *m*×78, where *m* is number of features. Once the input dimension is reduced, a classification network is trained to find the optimal weight matrix. A perturbed projection matrix is then computed by simply solving a linear system as described in the “Description of the model” section.

### Data description

**Fig. 1:**
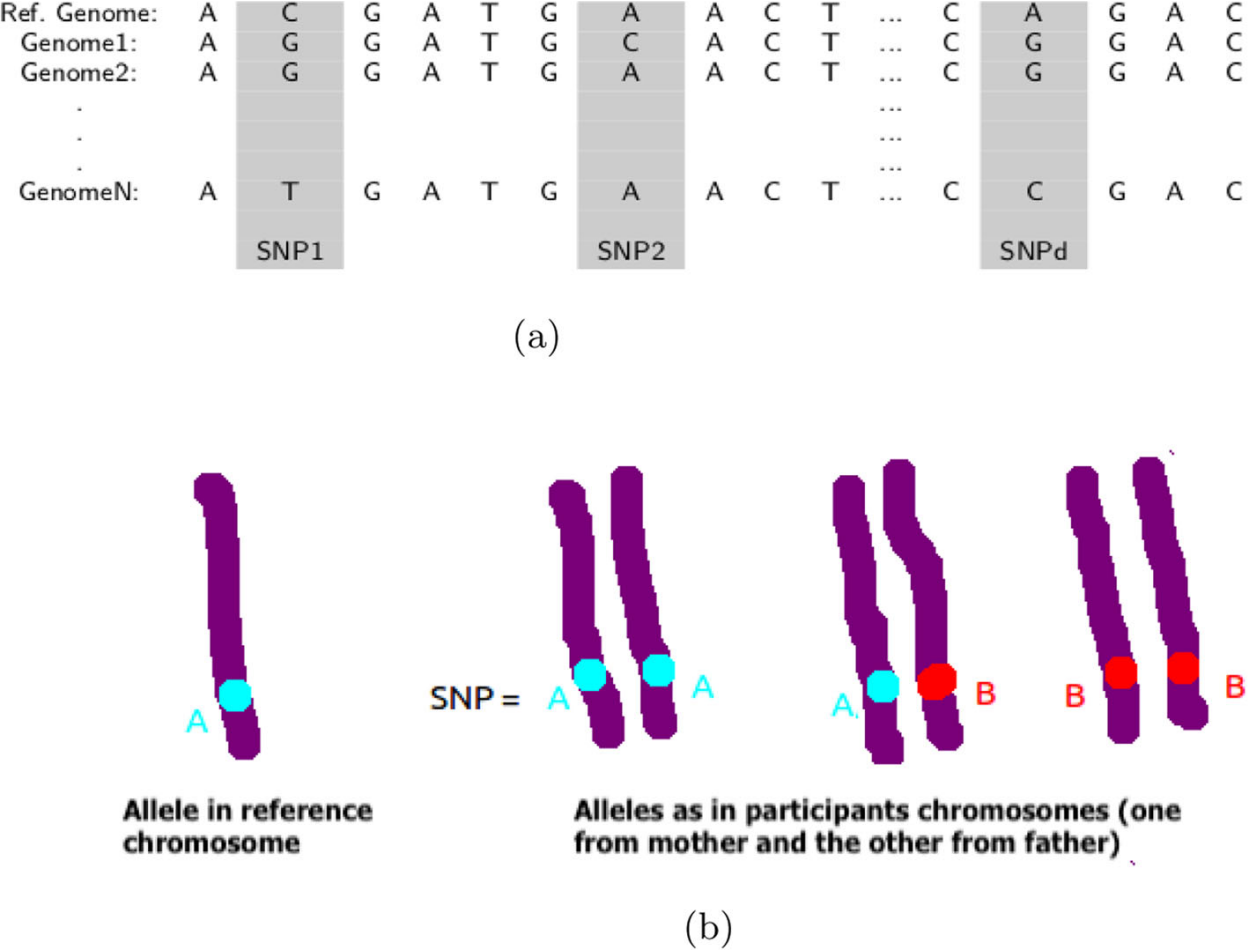
**a** Illustation of *SNPs*, **b** Three possible values taken by *SNPs*

1000 Genomes Project set up in 2008 [52], is an international research consortium which aims to produce a detailed catalog of humans genetic variations, from approximately one thousand volunteers from different ethnic groups, with frequencies larger than 1%. It is the first project to sequence the genome of a large number of people from different populations, regions and countries. Data made available to the international community comprises *SNP* profiles of the volunteers (see Fig. 1a), which is a vector where the coordinates are the values taken in a fixed position in the genome sequence (*homozygous reference*, *heterozygous* or *homozygous alternate*).

At each locus (fixed position in the genome sequence), an *SNP* is represented by its genotype that takes three possible values for a diploid organism: AA for *homozygous reference*, AB for *heterozygote* and BB for *homozygous alternate* (see Fig. 1b). The *homozygous reference* corresponds to a locus where the two base pairs inherited from the parents are identical to the one in the reference genome, the *heterozygous* corresponds to a locus where the two base pairs found are different and *homozygous alternate* refers to a locus where the two base pairs found are identical and different from the reference base pair.

Before any further processing, these values were converted into numerical values, e.g., AA=0, AB=1 and BB=2, using the tool Plink [53].

The dataset taken as input for the model is a matrix $X \in \mathbb {R}^{3450\times 315345}$. The rows of the matrix correspond to individuals (1000Genome’s volunteers), the columns correspond to *SNPs* positions, and the elements are 0, 1 or 2 (corresponding to the three possible values taken by an *SNP*). 3450 is the number of individuals sampled worldwide from 26 population groups from the 5 continents (see Appendices) and 315345 is the number of included features (*SNPs* positions).

We use a classification Neural Network composed of an input layer, an output layer and two hidden layers with 100 neurons. This neural network is constructed using Keras and Tensorflow open source libraries. Given the input matrix *X*, the output of the model is a vector $Y \in \mathbb {R}^{c}$ whose components correspond to the population groups (26 classes in the used example). A relu activation function is used in the two hidden layers followed by a softmax layer to perform ancestry prediction.

### Singular value decomposition

Before applying *SVD*, input data set is divided into two sets, the training set and the test set. *SVD* takes as input the training set matrix transpose denoted by $X^{T}\in \mathbb {R}^{m\times n}$(*m*>*n*) with *rank*(*X*)=*r* and decomposes it into a product of three matrices [54]; two orthogonal matrices $U \in \mathbb {R}^{m\times m}$ and $V \in \mathbb {R}^{n\times n}$ and a matrix $\Sigma =diag(\sigma _{1},\sigma _{2},\ldots,\sigma _{n})\in \mathbb {R}^{m\times n}$, *σ*_*i*_>0 for 1≤*i*≤*r*, *σ*_*i*_=0 for *i*≥*r*+1, such that

$$X^{T}=U\Sigma V^{T}=\sum\limits_{i=1}^{r}U_{i}\Sigma_{i}V^{T}_{i}. $$

The first *r* columns of the orthogonal matrices *U* and *V* are, respectively, the right and the left eigenvectors associated with the *r* nonzero eigenvalues of *X*^*T*^*X*. *U*_*i*_,*V*_*i*_ and *Σ*_*i*_ are, respectively, the *i*th column of *U*, *V* and *Σ*. The diagonal elements of *Σ* are the nonnegative square roots of the *n* eigenvalues of *X*^*T*^*X*.

The dimension of the input matrix *X* is then reduced by projecting it onto a space spanned by {*U*_1_,*U*_2_,…,*U*_*k*_}, the top *k* (*k*≤*r*) singular vectors of *X*. Given a set of samples *x*_1_,*x*_2_,…,*x*_*N*_ of dimension *m*, the projection matrix *U*^*k*^ whose columns are formed by the *k* first singular vectors of *X* must minimize

$$\sum\limits_{i=1}^{N}\lVert{P(x_{i})-x_{i}}\rVert_{2}^{2}=\sum\limits_{i=1}^{N}\lVert{x_{i}U^{k}-x_{i}}\rVert_{2}^{2} =\lVert{XU^{k}-X}\rVert_{2}^{2}, $$

where *P* is the projection defined by :

$$\begin{array}{@{}rcl@{}} P &: &\mathbb{R}^{m}\longrightarrow \mathbb{R}^{k}\\ &&x \longrightarrow x'=xU^{k} \end{array} $$

.

The input data in reduced dimension is denoted by *X*^′^=*X* *U*^*k*^.

### Description of the model

**Fig. 2:**
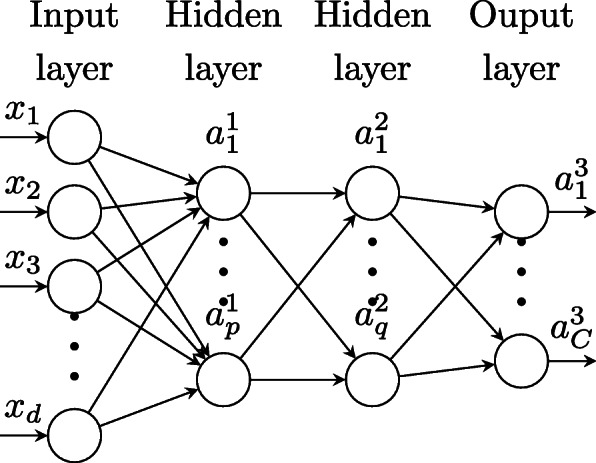
Classification network(MLP)

Let’s consider a *L* hidden layers of a Multi-Layer Perceptron(*MLP*), in which *n* input training samples *X*={**x**_1_,**x**_2_,…,**x**_*n*_} are labeled, i.e., for each input **x**_*i*_, the corresponding output by the model is known and denoted *y*_*i*_ or *Y*(**x**_*i*_). *Y* is a vector that contains all the labels. A *MLP* can be described as follows:

1 $$\begin{array}{*{20}l} a^{(l)}_{j}&=\phi\left(z^{l}_{j}\right), \end{array} $$

2 $$\begin{array}{*{20}l} z^{l}_{j}&=\sum\limits_{i}w^{l}_{ij}a^{(l-1)}_{i} + b^{l}_{j}=\mathbf{a}^{(l-1)}.\mathbf{w}^{l}_{j}+b^{l}_{j}, \end{array} $$

where $z^{l}_{j},b^{l}_{j}$ and $a^{l}_{j}$$\left (a^{0}_{j}=x_{j},\,\, \text {for an input}\,\, \mathbf {x}=(x_{1}\,x_{2}\,\ldots x_{d})^{T} \right)$ are the *j*th hidden unit, bias term and activation function of layer *l*, respectively. $w^{l}_{ij}$ is the weight that links the *i*th unit of the (*l*−1)th layer to the *j*th unit of the *l*th layer. $\mathbf {w}^{l}_{j}$ and **a**^(*l*−1)^ are, respectively, the incoming weight vector to the *j*th neuron of layer *l* and the output vector of *(l-1)*th layer, *ϕ* is any activation function. Learning the model consists in finding all the parameters **w**_*j*_ and *b*_*j*_ so that the output **a**^*L*^ from the model approximates the true output vector **y**(**x**), for all training inputs **x**.. For simplification, we consider that there are no bias terms $b^{l}_{j}$ or simply we consider it as an additional component of $\mathbf {w}^{l}_{j}$ and denote by *W*^*l*^ the matrix whose columns are the vectors $\mathbf {w}^{l}_{j}$ (Fig. 2).

**Fig. 3:**
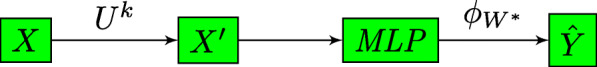
Classification network before input perturbation(*MLP*)

Due to the high dimension of the input data, the proposed approach consists to first project the original data onto a lower dimensional space using *SVD*. Once the dimension of the input data is reduced, a multilayer perceptron (*MLP*) classification network is constructed in three steps:

- **Step 1** **Learning the weight matrix*W* :** First, a classification network(see Fig. 3) is trained to find *W*^∗^, the optimal weight by solving:
  3 $$ W^{*}=\underset{W}{arg\,min}\,\,C_{W}(X',Y).   $$
  Where $C_{W}(X',Y)=||\phi _{W}(X')-Y||_{2}^{2}$ and $\hat {Y}=\phi _{W^{*}}(X')$. *ϕ*_*W*_ is the output activation function for the weight matrix *W*. *Y* represents the true classification labels.
- **Step 2** **Input matrix perturbation*X***^***′***^**:** Once the classification network is sufficiently trained, its weight matrix *W*^∗^ is fixed and the training input matrix *X*^′^ is perturbed to find *X*^′^^∗^ solution of the following problem :
  4 $$ X'^{*}=\underset{Z}{arg\,min}\,\,C_{W^{*}}(Z,Y),   $$
  To perturb the input data, we use an iterative version of *FGSM*(see Appendices: Fast gradient sign method) that adds a non random noise whose direction is opposed to the gradient of the loss function.
- **Step 3** **Projection matrix perturbation*U***^***k***^**:** After finding the optimal perturbation *X*^′^^∗^, we look for a perturbed projection matrix *U*^*k*^^∗^ by solving the following linear system :
  5 $$ {U^{k}}^{*}=\underset{V}{arg\,min}\,\,||XV-X'^{*}||_{2}^{2}.   $$
  Where *X* is the original training matrix and *V* any matrix, with the same size as *U*^*k*^. After the three construction steps, the output of the *MLP*, is $\hat {\hat {Y}}=\phi _{W}^{*}(X'^{*})$. Once *U*^*k*^^∗^ is calculated, we project the original test set on the latter to evaluate the performance of the classification network.

**Fig. 4:**
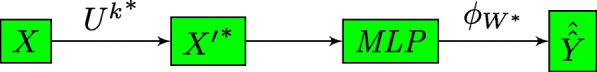
Classification network after inputs perturbation(*MLP-IP*)

It is worth noting that, after recovery of the perturbed inputs, the classification network (see Fig. 4) can be re-trained or tested with the fixed weight matrix *W*^∗^(in **Step 2**). From Step 1 and after having solved the system (4), the input matrix *X* can be perturbed by solving :

6 $$ {U^{k}}^{*}=\underset{V}{arg\,min}\,\,||\phi_{W^{*}}(XV)-Y||_{2}^{2}.   $$

But the high dimensionality of input data makes the non-linear optimization problem difficult to solve and the results less accurate.

## Results

In this section, the obtained results using the proposed method are reported and its performance is compared to that of the once recommended in [33] (the **Per class histogram**, see Appendices: Thin parameters for fat genomics, Table 2).

### Proposed method

In the table below, we summarize the accuracy of the classification with respect to the number of modes (principal components) *k* chosen between 20 and 1000.

Table 1 represents in the second column (resp. third column) the results obtained by the classification network before (resp. after) input perturbation. After input perturbation, the training model can be evaluated using the fixed weight matrix (in the third column) as well as re-trained (in the last column). It is clear from the above results that input perturbation has significantly reduced misclassification.

**Table 1:** Results obtained by the classification network, before and after inputs perturbation

*k*	*MLP*	*MLP*-*IP*	*MLP*-*IP*-*R*
10	76.46	84.63	87.82
20	84.84	92.02	92.29
50	91.88	96.23	95.71
100	92.75	95.21	94.55
200	92.89	95.65	95.68
500	93.93	94.92	95.44
1000	94.05	94.34	94.02

To illustrate the effectiveness of the proposed method, we display the confusion matrix of our classification network to see the effect of input perturbation.

In Fig. 4a (before input perturbation), we observe high classification errors between some population groups such as Chinese Dai in Xishuangbanna and the Kinh in Ho Chi Minh City; Indian Telugu in the UK and Sri Lankan Tamil in the UK; or British in England and Scotland and Utah Residents (CEPH) with Northern and Western Ancestry. Figure 4b shows how our approach has reduced these misclassifications, particularly the classification error between the CDX and KHV classes from **0.95%** to **0.05%**.

**Fig. 5:**
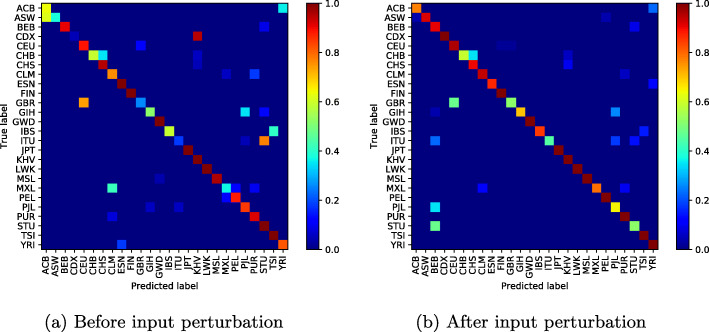
10 mode-confusion matrix

**Fig. 6:**
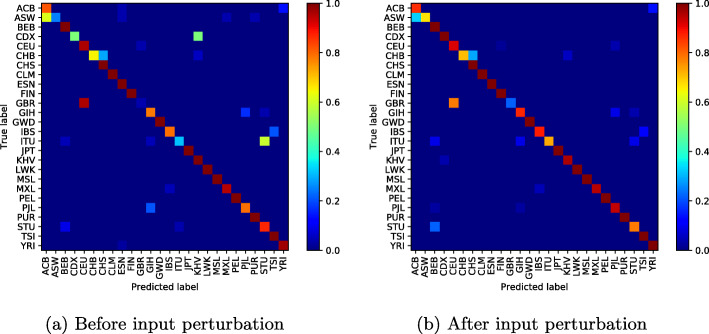
20 mode-confusion matrix

**Fig. 7:**
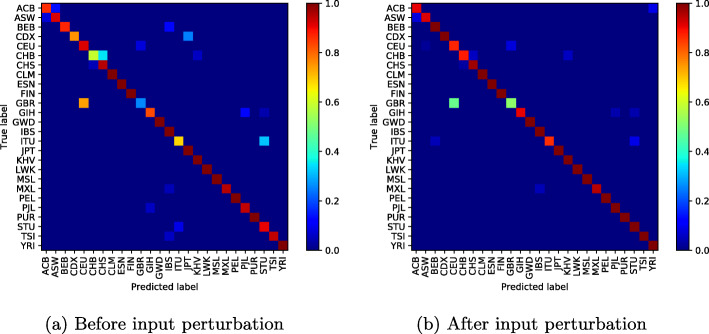
50 mode-confusion matrix

**Fig. 8:**
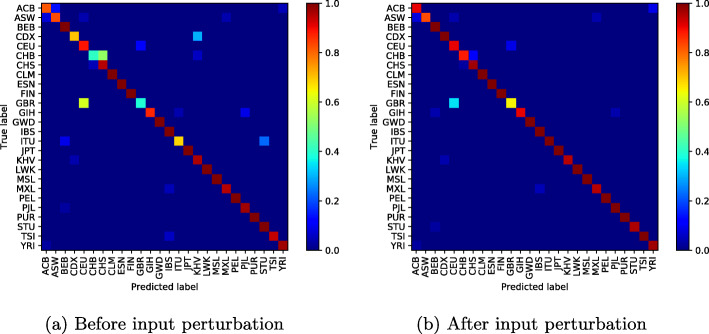
100 mode-confusion matrix

**Fig. 9:**
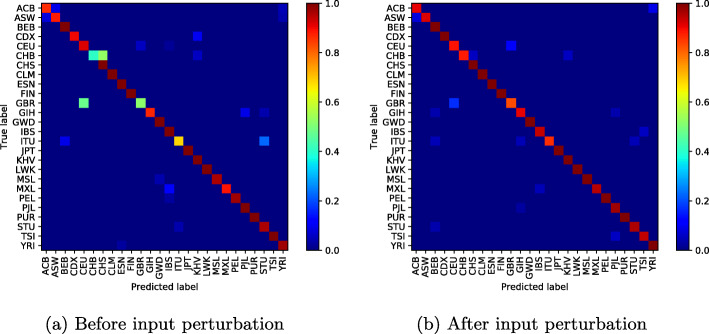
200 mode-confusion matrix

However, as the number of modes increases and the classification errors decrease, one can notice throughout our experiences a weak classification error between the British ethnic groups in England, Scotland and Utah Residents (CEPH) with Northern and Western Ancestry, who appear to be genetically very similar (Figs. 5, 6, 7, 8 and 9).

### Per class histogram

**Fig. 10:**
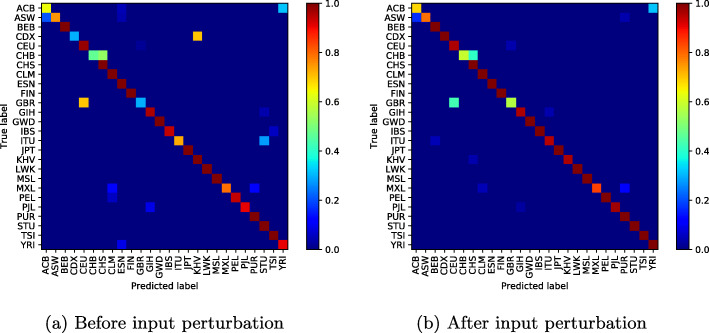
Per class histogram confusion matrix

In Fig. 10, we present confusion matrices obtained by per histogram embedding methods and Per class histogram embedding input perturbation. Perturbing per class embedding input reduced misclassification errors and allowed the classifier to reach **94,49%** of accuracy.

## Discussion

Deep learning application to high-dimensional genomic data, such as *SNPs* is more challenging. In order to deal with problems of high dimensionality, many efforts have been made. In [11], the authors proposed to learn the feature presentations using a Neural Network followed by another classification network. Unsupervised clustering or Deep Autoencoder is jointly trained with a classification network [13, 32, 33, 55]. However, these methods are generally applied to datasets with relatively small features where, the computational cost increases linearly with the number of features and they require more training samples to converge. When Autoencoder network was trained jointly with the classification network on the used dataset, the best accuracy obtained was 85.36%. In addition to the high dimensionality of the data, there is another challenge related to the high genetic similarity between certain population groups. To mitigate these difficulties, the proposed method reduces the dimension of the input data using SVD algorithm. However, the SVD algorithm extracts linear combinations of features from the input data and fails to take into account the genetic similarity between some population groups as shown in Figs. 4a-10a. To improve these results, the SVD projection matrix is modified to minimize the training loss function of the classification network using FGSM algorithm. The FGSM algorithm allowed us to find the best representation of the input features in SVD projection space. This new representation makes the classification network more robust to small variations in the input and takes into account the genetic similarity between different populations, as shown in the last two columns of Table 1 and Figs. 4b-10b. We are not limited to the SVD algorithm, when Per class histogram is used to reduce the dimension of the input data, the proposed perturbation has significantly reduced classification errors.

The proposed method has achieved its best results when the input features were reduced from 300M to 50, which means that the number of free parameters of the classification network has reduced by a factor of 6000. This method outperforms previous work (see Appendices: Thin parameters for fat genomics) in term of accuracy and the number of free parameters required by the model. For future work, we expect to improve this method by using different targeted attacks algorithms with other dimensionality reduction techniques.

## Conclusion

In this work, we proposed a New Neural Network method for the prediction of individual ancestry from *SNPs* data. To deal with the high dimensionality of the *SNPs* data, our approach first uses *SVD* to reduce the dimensionality of its inputs, then train a classification network and then reduce prediction errors by perturbing the input data set.

The obtained results showed how input perturbation reduced classification errors despite genetic similarities between some ethnic groups. With such accuracy in the task of predicting genetic ancestry, this method will make it possible to deal with more complex problems in the healthcare field. We therefore, intend to apply our method to gene expression profiles as well as *SNPs* data in order first to predict and then prevent the development of patients genetic diseases.

## Appendices

### Fast gradient sign method

*FGSM* ([40]) : uses the gradient of the loss function to determine in which direction the input data features should be changed to minimize the loss function :

$$x'=x-\epsilon sign(\nabla_{x} C_{W}(x,y)), $$

*ε* is a tunable parameter. Iterative Fast Gradient Sign Method (*IFGSM*) consists in adding the perturbation iteratively [47]. In our context, given any input training sample *z*_*i*_ (a row of the training input matrix *X*) and its corresponding one-hot label *y*_*c*_, we pertub it in the direction of the input space which yields to the hightest decrease of the loss function $\phantom {\dot {i}\!}C_{W^{*}}$, using the Targeted Iterative Fast Gradient Sign Method (*IFGSM*) given by the formula :

$${z_{i}}^{(m)}={z_{i}}^{(m-1)}-\epsilon sign\left(\nabla_{z_{i}} C_{W^{*}}\left({z_{i}}^{(m-1)},y_{c}\right)\right), $$

where *m*=1,…,*M*,*z*_*i*_^(0)^=*z*_*i*_, *M* is the number of iterations and *z*_*i*_^∗^=*z*_*i*_^(*M*)^ the perturbed version of *z*_*i*_. After perturbation, the rows of the matrix *X*^′^^∗^ are composed of *z*_*i*_^∗^ for *i*=1,…,*n*. Where *n* is the number of training samples.

### genome project legends

#### Population ethnicity legend

**ACB**: African Caribbeans in Barbados; **ASW**: Americans of African Ancestry in SW USA; **BEB**: Bengali from Bangladesh; **CDX**: Chinese Dai in Xishuangbanna; **CEU**: Utah Residents (CEPH) with Northern and Western Ancestry; **CHB**: Han Chinese in Bejing; **CHS**: Southern Han Chinese; **CLM**: Colombians from Medellin; **ESN**: Esan in Nigeria; **FIN**: Finnish in Finland; **GBR**: British in England and Scotland; **GIH**: Gujarati Indian from Houston; **GWD**: Gambian in Western Divisions in the Gambia; **IBS**: Iberian Population in Spain; **ITU**: Indian Telugu from the UK; **JPT**: Japanese in Tokyo; **KHV**: Kinh in Ho Chi Minh City; **LWK**: Luhya in Webuye; **MSL**: Mende in Sierra Leone; **MXL**: Mexican Ancestry from Los Angeles; **PEL**: Peruvians from Lima; **PJL**: Punjabi from Lahore; **PUR**: Puerto Ricans; **STU**: Sri Lankan Tamil from the UK; **TSI**: Toscani in Italia and **YRI**: Yoruba in Ibadan.

#### Geographical region legend

**AFR**: African; **AMR**: Ad Mixed American; **EAS**: East Asian; **EUR**: European and **SAS**: South Asian.

### Thin parameters for fat genomics

We represent in Table 2, different results from [33].

**Table 2:** Obtained results by [33]

Model & Embedding	Mean Misclassif. Error. (%)	# of free param.
Basic	8.31±1.83	31.5M
Raw end2end	8.88±1.41	21.27K
Random Projection	9.03±1.20	10.1K
SNP2Vec	7.60±1.28	10.1K
Per class histograms	7.88±1.40	7.9K
Basic with reconstruction	7.76±1.38	63M
Raw end2end with reconstruction	8.28±1.92	227.3K
Random Projection with reconstruction	8.03±1.0.3	20.2K
SNP2Vec with reconstruction	7.88±0.72	20.2K
Per class histograms with reconstruction	7.44±0.45	15.8K

## Data Availability

The dataset used in this work is freely available(http://ftp.1000genomes.ebi.ac.uk:21/vol1/ftp/release/20130502/supporting/hd_genotype_chip/) and the open source libraries used be can found here (https://www.tensorflow.org/guide/keras/overview)
